# Pathophysiological Role of Synovitis in Hemophilic Arthropathy Development: A Two-Hit Hypothesis

**DOI:** 10.3389/fphys.2020.00541

**Published:** 2020-06-09

**Authors:** Ilenia Calcaterra, Gabriella Iannuzzo, Francesco Dell’Aquila, Matteo Nicola Dario Di Minno

**Affiliations:** ^1^Department of Clinical Medicine and Surgery, School of Medicine and Surgery, University of Naples Federico II, Naples, Italy; ^2^Department of Translational Medical Sciences, University of Naples Federico II, Naples, Italy

**Keywords:** hemophilic arthropathy, cytokines, inflammation, synovitis, pathophisiology

## Abstract

Despite an increasing access to prophylaxis with clotting factor concentrates, arthropathy still represents the main chronic complication of hemophilia. Whereas previous studies described hemophilic arthropathy (HA) as a degenerative arthropathy, somehow resembling osteoarthritis (OA), most recent evidence suggests that complex inflammatory and immunologic mechanisms are also involved in the pathophysiology of HA. In the present review, we described available data on major mechanisms leading to arthropathic changes in patients with hemophilia, with a specific focus on the role of synovium. The presence of hemosiderin in the joint space induces synovium proliferation, thus leading to formation of several lytic enzymes determining chondrocytes apoptosis and proteoglycans levels reduction. This leads to a direct joint “chemical” damage representing early damages in the pathogenesis of HA (first hit). In parallel, synovial membrane and synovial endothelial cells become a dynamic reservoir of inflammatory cells and mediators, and propagate the inflammatory response (second hit), switching the process from a chemical damage to an inflammatory damage. Overall, consistent data pointed out synovitis as the keystone in HA pathophysiology. This opens novel potential therapeutic targets in this clinical setting.

## Introduction

Hemophilia is a genetic X-linked coagulative disorder caused by the deficiency of coagulation factor VIII (hemophilia A) or coagulation factor IX (hemophilia B). Incidence is 1/5000 for hemophilia A and 1/30000 for hemophilia B (Acharya, 2012). Affected individuals report an increased bleeding risk, with joints being the anatomical site most often involved (Di Minno et al., 2016). All joints can be potentially involved, but hemarthrosis usually occurs in large synovial joints (knee, ankles, and elbows), thus progressively leading to a severe and disabling arthropathy (Arnold and Hilgartner, 1977).

Although a more severe bleeding phenotype has been recognized in patients with severe hemophilia A (<1% FVIII activity), some data showed that we can observe a significant incidence of HA also in patients with moderate hemophilia (2–5% FVIII activity) (Di Minno et al., 2013).

While an effective prophylactic factor replacement therapy considerably reduced joint bleeding episodes, some signs of hemophilic arthropathy (HA) are still reported by 25–30% of patients, even in highly developed countries (Arnold and Hilgartner, 1977; Manco-Johnson et al., 2007; Wojdasiewicz et al., 2018). Thus, arthropathy still represents the main chronic complication of hemophilia.

Several previous studies described HA as a degenerative arthropathy, somehow resembling osteoarthritis (OA) (Pulles et al., 2017). In contrast, most recent evidence suggests that complex inflammatory and immunologic mechanisms are also involved in the pathophysiology of HA. The aim of the present review is to describe available data on major mechanisms leading to arthropathic changes in patients with hemophilia, focusing on the role of synovial tissue.

## Synovial Tissue

In physiologic conditions, the synovial tissue is involved in the production of synovial fluid that fills articular cavity and lubricates bony structures to ensure a correct articular excursion. On the other hand, synovial tissue has a pivotal role in pathogenesis of HA (Arnold and Hilgartner, 1977).

Indeed, the synovial membrane, a specialized connective tissue, consists of two layers, the intima and the sub-intima, with a small amount of hyaluronic acid between layers. The intima is relatively acellular and consists of two types of synoviocytes: type A (monocyte-macrophage cell-like) and type B (fibroblast-like). The sub-intima is composed of lymphatic vessels and is highly vascularized (Smith, 2011). Although the presence of numerous capillaries in the synovial tissue is of great importance for physiologic functions, unfortunately they are also the source of joint bleeds (Jansen et al., 2008).

## Iron Chemical Damage in Synovitis (Figure 1)

When a hemarthrosis occurs, blood-derived iron (hemosiderin) deposition determines a chemical damage to the synovial tissue leading to activation of inflammatory and anti-apoptotic patterns. In a study conducted on murine models of hemarthrosis, an iron-induced chemical damage was demonstrated, also emphasizing the pathogenic role of iron-derived metabolites [Ferroportin (an iron cell exporter); Hepcidin (regulator of FPN); Hemoglobin scavenger receptor (CD163); Heme carrier protein 1 (heme cell importer); Feline leukemia virus subgroup C (heme cell exporter)] (Nieuwenhuizen et al., 2013). These data have been confirmed in a study comparing synovial histological sections of patients affected by rheumatoid arthritis (RA), OA, and HA. Nuclear and cytoplasm expression of iron-derived metabolites was much more abundant in synovial tissue of hemophilic patients as compared to OA and RA, thus suggesting a crucial role in pathophysiology of HA (Nieuwenhuizen et al., 2013). In particular, hemosiderin deposition within synoviocytes and the presence of iron metabolites are associated with the production of reactive oxygen species (ROS) via the Haber–Weiss/Fenton reaction (Fe^2+^ + H_2_O_2_ → Fe^3+^ + OH^–^ + OH^–^) (Manco-Johnson et al., 2007; Valentino, 2010; Blobel et al., 2015). In turn, the thin synovial membrane becomes a hypertrophic and villous membrane, via induction of DNA-synthesis and cell proliferation. In fact, hemosiderin inhibits synovial cell apoptosis by stimulating the amplification of myelocytomatosis viral oncogene (c-MYC) (a proto-oncogene associated with cell proliferation) and of mouse double minute 2 (MDM2) homolog (a protein that targets the tumor suppressor gene p53) (Hakobyan et al., 2004; Pulles et al., 2017).

**FIGURE 1:**
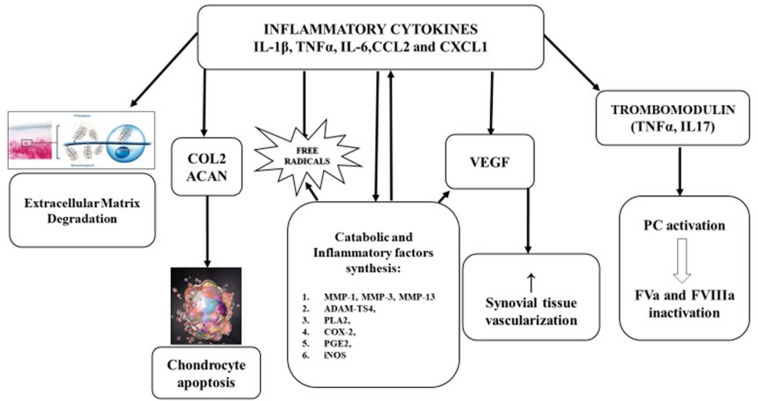
Pathophysiology of hemophilic arthropathy. Type A synoviocytes, after incorporating iron, produce and relapse inflammatory cytokines (IL-1β, IL-6, TNFα) and chemokines (CCL2, CXCL1), leading to migration of polymorphonuclear cells and later, of monocytes and lymphocytes. The consequent inflammatory response promotes:
•Extracellular matrix degradation.•Inhibition of proteoglycan and collagen type II (COL2) synthesis by chondrocytes and induce apoptosis.•Expression of metalloprotease (MMP-l, MMP-3, MMP-13, andADAMTS4) that have a pivotal role in catabolic joint processes.•Expression of cyclooxygenase 2 (COX-2) and prostaglandin E2 (PGE2) involved in development and maintenance of inflammatory process.•Neo-angiogenesis, stimulating, both locally and systemically, the release of growth factors like vascular-derived endothelial growth factor (VEGF).•Liberation of trombomodulin (TM) by inflammatory cells, TM binds, then activates protein C (PC) inducing factor V (FVa) and FVIIIa degradation.

These findings are consistently confirmed by results of the study by Wen et al. (2002) showing that iron is involved in the modulation of the expression of c-MYC and MDM2 homolog, leading to proliferation of the synovium.

Hypertrophic synovium produces several lytic enzymes that, by means of a “chemical damage,” induce chondrocytes apoptosis and proteoglycans level reduction. According with these pathophysiological mechanisms, a recent study conducted on hemophilia murine models in which hemarthroses were artificially induced showed that HA-related signs of degenerative manifestations quickly appear after exposition to blood products. In fact, histological section evaluation highlighted that synovitis was developed within 24 h, whereas cartilage and bone damage became manifest within 48–96 h. This could suggest a direct influence of blood on these processes besides indirect effect of inflammation (Christensen et al., 2019).

Overall, early damages secondary to iron-mediated chemical injury could represent the first step in the pathogenesis of HA (*FIRST HIT*) (Roosendaal et al., 1999).

In parallel, iron plays a crucial role in the induction of the expression of several pro-inflammatory cytokines, including interleukin 1 beta (IL-1β), tumor-necrosis factor alpha (TNFα), and interleukin 6 (IL-6) (Melchiorre et al., 2017).

In detail, type A synoviocytes, after incorporating iron, produce inflammatory cytokines (IL-1β, IL-6, TNFα), in turn inducing migration of polymorphonuclear cells and, later, of monocytes and lymphocytes. This leads to a self-maintaining cycle further increasing inflammatory response and inducing an enhanced angiogenesis (Lafeber et al., 2008; Agapidou et al., 2016). Indeed, the inflamed and hypertrophic synovium has an enhanced oxygen demand, stimulating both locally and systemically the release of growth factors like vascular-derived endothelial growth factor (VEGF), thus promoting neo-angiogenesis (Pulles et al., 2017).

These phenomena involving synovial tissue can induce a chronic inflammatory process mediated by cytokines and pro-angiogenic molecules, switching the process from a chemical damage to an inflammatory damage characterized by progressive synovial pannus growth and articular cartilage damage worsening (Valentino, 2010; Pulles et al., 2017).

Thus, synovial membrane and synovial endothelial cells become an active reservoir of inflammatory cells and mediators, and propagate the inflammatory response (*SECOND HIT*).

Currently, IL-1β and TNFα are the most widely studied inflammatory cytokines involved in the pathogenesis of HA (Pulles et al., 2017; Wojdasiewicz et al., 2018).

## Role of Inflammatory Cytokines (Figure 1)

### IL-1β

IL-1β is one of the main regulators of inflammatory response. IL-1β induces catabolic processes in synovial joint both directly, acting on cell, and amplifying inflammatory processes through activation of transduction signal pathways.

The “inflammasome” is a crucial factor regulating the maturation and secretion of pro-inflammatory IL-1 (Dutra et al., 2014; Srivastava, 2015). After interacting with its receptor, IL-1 leads to the activation of nuclear factor kappa-light-chain-enhancer of activated B cells (NF-κB) transcriptive factor and other transcriptive factors such as c-Jun N-terminal kinase (JNK) and p38 mitogen-activated protein kinases (p38MAPK). As a result, there is an increased expression of various genes responsible for the synthesis of enzymes, adhesion molecules, or inflammatory mediators including cytokines and chemokines (Wojdasiewicz et al., 2018). This is in line with observations of the role of NFkB in synovitis development and cartilage degeneration in OA and RA (Melchiorre et al., 2012; Pulles et al., 2017).

Consistently confirming the involvement of IL-1β in the pathophysiology of HA, some authors (Tagariello and di Giovine, 1996; Valentino, 2010; Dutra et al., 2014; Srivastava, 2015) documented markedly elevated IL-1β levels in histological section of the synovial membranes collected during synovectomy or joint replacement from HA patients as compared to patients without hemophilia (Roosendaal et al., 1999).

On the other hand, IL-1β can also increase transferrin-bound iron uptake into type B synoviocytes which leads to deposition of hemosiderin and IL-1β autocrine secretion and, consequently, to development of chronic synovitis (Telfer and Brock, 2004).

### TNFα

TNFα is a member of tumor necrosis factor superfamily and plays a crucial role in HA pathophysiology. TNFα also induces catabolic processes in synovial joint and directly regulates intra-articular levels of FVIIIa modulating expression of thrombomodulin (TM) (Aggarwal et al., 2012; Wojdasiewicz et al., 2018).

In particular, TNFα inhibits proteoglycan and collagen type II (COL2) synthesis by chondrocytes. It can induce the expression of metalloprotease (MMP-1, MMP-3, MMP-13, and ADAMTS4) that have a pivotal role in catabolic joint processes (Wojdasiewicz et al., 2018).

On the other hand, TNFα has a direct role in increasing the risk of bleeding recurrence. TNFα is associated with a substantial reduction of TM synthesis by synoviocytes, due to a huge liberation of TM into the synovial fluid induced by an intensive action of neutrophils and cytokines on synovial cells. Additionally, a recent study shows that synovial fluid TM levels were more elevated in patients with HA (56 ± 25 ng/mL) as compared to healthy controls (39 ± 21 ng/mL). In physiologic conditions, TM binds thrombin in a 1:1 stoichiometric ratio, then activates protein C (PC) (Dargaud et al., 2012). PC is a zymogen which belongs to a group of proteins which inhibits coagulation by inducing factor V (FVa) and FVIIIa degradation (Anastasiou et al., 2012; Wojdasiewicz et al., 2018). This interaction between inflammatory mediators and hemostasis components might explain why the hemorrhagic process can sometimes be sustained, despite the FVIII replacement therapies.

Thus, IL-1β and TNFα, triggering and amplifying inflammatory damage and its consequences on joint, represent the cornerstone in pathophysiology of HA. Moreover, TNFα has an important and documented role in the regulation of hemostatic balance of joint in patients with HA.

Moreover, recent evidence showed an increased synovial tissue expression of the TNFα/TNF receptor (TNF-R) system. The activation of this system could represent a crucial mediator of synovial proliferation and a potential novel target for therapy (Manetti et al., 2019).

Furthermore, a recent study showed that similarly to OA and RA, patients with HA exhibit increased levels of progranulin (PGRN), a molecule known for its protective role toward TNFα catabolic effects (Kotela et al., 2018). This evidence could open future hypotheses on its potential role as a serum-maker for monitoring disease activity.

## Challenges in the Treatment of Ha (Figure 2)

Clotting factor replacement therapy represents the stronghold in hemophilia treatment but new knowledge about the pathophysiology of HA leads to new issues concerning potential therapeutic targets. An alternative potential approach is represented by the reduction of intra-articular iron deposition by means of iron chelators (deferoxamine, deferasirox) to stop the process at very early stages.

**FIGURE 2:**
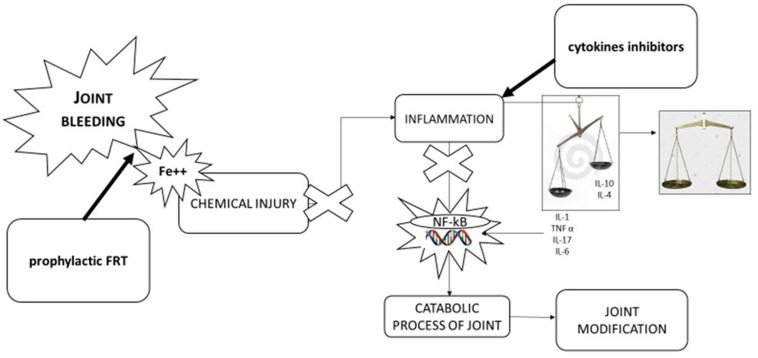
Pathophysiology of hemophilic arthropathy and potential therapeutic approach. Joint bleeding and consequent chemical injury due to iron exposition is the “*primwn movens*” in development of hemophilic arthropathy leading to inflammation and alteration of natural balance between inflammatory cytokines (IL-1β, IL-6, TNFα, IL-17) and anti-inflammatory cytokines (IL-10, IL-4). Tins leads activation of nuclear transcription factor [nuclear factor kappa-light-chain-enhancer of activated B cells (NF-KB)] inducing catabolic process of joint and in turn joint modification. Therapeutic approaches can be direct to stop key point of pathological process: first factor replacement to stop joint bleeding, using anti-inflammatory drugs [cyclooxygenase 2 (COX-2) inhibitors, monoclonal antibodies anti-TNFα, and anti-IL-1β] to balance cytokines pathway.

A further option is represented by anti-inflammatory therapy using cyclooxygenase 2 (COX-2) inhibitors, monoclonal antibodies anti-TNFα and anti-IL-1β with the aim to avoid the self-maintaining inflammatory cycle. Evidence showed that COX-2 inhibitors (celecoxib and rofecoxib) are safe and effective in treating chronic synovitis and joint pain, and currently represent a potential choice to treat pain in hemophilia patients (Rattray et al., 2006; Tagliaferri et al., 2018; Santoro et al., 2020). In 2013, Melchiorre et al. reported data about a drastic reduction of joint bleeding in three patients treated with an anti-TNFα monoclonal antibody. These interesting findings are potentially due to the cross-talk between inflammation and hemostasis mediated by TM inhibition (Melchiorre et al., 2014). On the other hand, in another study on human chondrocyte cells cultures exposed to human blood cells (as a model of joint bleeding), the addition of monoclonal anti-TNFα antibodies did not reduce chondrocytes apoptosis and did not improve proteoglycans synthesis. On the contrary, the addition of monoclonal antibodies against IL-1β reduced chondrocytes apoptosis and enhanced proteoglycans synthesis. These findings suggest that TNFα inhibition, although able to reduce joint bleeding, could not have a direct positive effect on joint deterioration, whereas promising effects on cartilage could be expected using anti IL-1β monoclonal antibodies (van Vulpen et al., 2015).

Furthermore, a recent study conducted on murine models showed that the inhibition of iRhom2/ADAM17/TNFα pathway by TNFα inhibition is able to prevent synovitis and bone degenerative damage development (Haxaire et al., 2018).

Overall, despite higher costs, monoclonal antibodies could provide further beneficial effects beyond the pure inflammatory effect and, therefore, could be considered as a valuable therapy instead of COX-2 inhibitors. However, further studies are needed to address this issue.

## Challenge in HA Monitoring

To identify early arthropathic changes for prevention of joint degeneration due to progression of HA is advised a periodic follow up of the joint status (Di Minno et al., 2017). The gold standard for evaluation of HA to date is MRI (Di Minno et al., 2016). Although MRI can be reputed highly sensitive to detect signs of disease activity and effective to perform a full evaluation of the joint surfaces, this exam presents some important limitation of execution in daily clinical practice. In fact, it is not possible to evaluate more than a joint for each exam, the time of exam performing is at least 30 min, and it is not comfortable for the patient. Furthermore, execution of MRI might require sedation for children and it is a high-cost technique (Di Minno et al., 2017).

In view of these limitations, rising interest has been reported in ultrasound (US) as a useful tool to evaluate joint status and to observe disease progression in hemophilic patients (Di Minno et al., 2016). The first practice to assess joint disease in hemophilic patients with US was performed by Wilson et al. (1987) in 38 patients with acute hemarthroses.

Ultrasound exam is able to detect and quantify most important biomarkers of disease activity such as joint effusion and synovial hypertrophy. Furthermore, one can find degenerative damages such as osteo-chondral changes through application of scoring scales (Martinoli et al., 2016a; Di Minno et al., 2017). In recent years, six scoring systems based on US have been proposed to quantify joint abnormalities in patients with hemophilia. Interestingly, all the US scores emphasize the role of synovitis detection as a maker of disease activity (Hermans et al., 2015; Martinoli et al., 2016b). Based on these, some recent UK guidelines defined the concept of “at risk joint” as a joint with synovitis (Hanley et al., 2017). On this hand, US examination by easily identifying synovitis could help guide physicians in the decision-making process of the optimal treatment for hemophilia patients.

Furthermore, identification of serum markers of disease activity (i.e., plasma levels of IL-1, TNFα, PGRN) could be a useful clinical tool to evaluate the severity of the joint disease and to guide the decision-making process for the treatment of each patient. Future studies should be designed to address this issue.

## Conclusion

At variance with previous evidence suggesting a purely degenerative nature of HA, several and consistent data clarified more complex underlying mechanisms, involving both degenerative alterations and inflammatory response, and pointing out synovitis as the keystone in HA pathophysiology. This opens novel potential therapeutic targets for HA and suggests a role of US for monitoring synovitis and guiding treatment tailoring in patients with hemophilia.

## Author Contributions

IC contributed to literature evaluation and manuscript drafting. GI contributed to literature evaluation and supporting in manuscript drafting. FD contributed to literature evaluation and supporting in manuscript drafting. MD coordinated and supervised manuscript drafting.

## Conflict of Interest

The authors declare that the research was conducted in the absence of any commercial or financial relationships that could be construed as a potential conflict of interest.
